# Erythropoiesis: insights from a genomic perspective

**DOI:** 10.1038/s12276-024-01311-1

**Published:** 2024-10-01

**Authors:** Hye Ji Cha

**Affiliations:** Department of Biomedical Science & Engineering, Dankook University, Cheonan, South Korea

**Keywords:** Haematopoietic stem cells, Differentiation

## Abstract

Erythropoiesis, the process underlying the production of red blood cells, which are essential for oxygen transport, involves the development of hematopoietic stem cells into mature red blood cells. This review focuses on the critical roles of transcription factors and epigenetic mechanisms in modulating gene expression critical for erythroid differentiation. It emphasizes the significance of chromatin remodeling in ensuring gene accessibility, a key factor for the orderly progression of erythropoiesis. This review also discusses how dysregulation of these processes can lead to erythroid disorders and examines the promise of genome editing and gene therapy as innovative therapeutic approaches. By shedding light on the genomic regulation of erythropoiesis, this review suggests avenues for novel treatments for hematological conditions, underscoring the need for continued molecular studies to improve human health.

## Genomic insights into erythropoiesis: unveiling key transcription mechanisms

Understanding how our bodies produce red blood cells, a process called erythropoiesis, is vital for treating blood disorders. Researchers have reviewed the stages of erythropoiesis, focusing on the genetic and molecular factors that influence the development of red blood cells. Erythropoiesis starts with stem cells in the bone marrow, which transform into several stages of red blood cell precursors before becoming mature, oxygen-carrying cells. The review emphasizes the role of specific growth factors, like erythropoietin, and transcription factors, proteins that activate or deactivate specific genes, in guiding these developmental stages. It also discusses how manipulating these factors in the lab can produce red blood cells outside of the body, a technique that could have therapeutic uses. By targeting the specific genes and pathways involved in red blood cell development, researchers can develop more effective treatments. This summary was initially drafted using artificial intelligence, then revised and fact-checked by the author.

## Introduction

Erythropoiesis, the process of red blood cell formation, is a fundamental pillar of human physiology, ensuring the efficient transport of oxygen from the lungs to tissues across the body. This complex process is initiated in the embryonic stage and continues throughout an individual’s life, adapting to the body’s changing needs from early development through adulthood. The journey of an erythroid cell from a pluripotent hematopoietic stem cell in the bone marrow to a fully mature, enucleated erythrocyte in the bloodstream exemplifies the remarkable complexity and precision inherent in human biology. This review explores the multifaceted stages of erythropoiesis, highlighting the genome-level and molecular mechanisms that govern the differentiation and maturation of erythroid cells. It delves into the roles of key transcription factors and epigenetic modifications in orchestrating these processes, the advancements in ex vivo erythroblast production for research and therapeutic purposes, and the consequences of pathway dysregulation leading to various erythroid disorders. By focusing on genomic-level regulation, this review sheds light on the intricate nature of erythropoiesis and provides insights into potential therapeutic targets for addressing erythroid disorders and advancing therapeutic strategies.

## Generation and maturation of erythroid cells

Erythropoiesis is an essential process for oxygen transport that occurs throughout life, from embryonic development to adulthood. This process evolves in complexity from primitive to definitive erythropoiesis, adapting to the changing requirements of the body from the embryonic stage, through fetal development, to lifelong maintenance^1^. Primitive erythroblasts, which are large nucleated cells, fulfill the oxygen requirements of early embryos but are soon replaced by more efficient erythrocytes produced through definitive erythropoiesis. In adults, erythropoiesis predominantly occurs in the bone marrow, where the process is regulated to yield mature, enucleated red blood cells that are optimal for oxygen carriage.

**Fig. 1:**
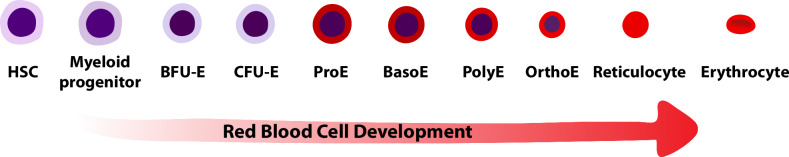
Schematic representation of erythropoiesis. HSC hematopoietic stem cell, BFU-E burst-forming unit-erythroid, CFU-E colony-forming unit-erythroid, ProE proerythroblast, BasoE basophilic erythroblast, PolyE polychromatic erythroblast, OrthoE orthochromatic erythroblast.

The widely accepted model posits that erythropoiesis is initiated when hematopoietic stem cells (HSCs) in the bone marrow differentiate into myeloid progenitors^2,3^. The progenitors further differentiate into erythroid progenitors, specifically the burst-forming unit-erythroid (BFU-E) and subsequently the colony-forming unit-erythroid (CFU-E). The CFU-E matures through several erythroblast stages—proerythroblast, basophilic, polychromatic, and orthochromatic erythroblast—synthesizing hemoglobin and undergoing cellular transformations in preparation for enucleation. The orthochromatic erythroblast expels its nucleus to form a reticulocyte, which enters the bloodstream and matures into a functional erythrocyte within approximately one week. The resultant biconcave, an enucleated cell-laden with hemoglobin, is primed for effective oxygen transport (Fig. 1).

The production of erythroblasts in liquid culture from CD34+ cells or stem cells represents a significant research avenue in regenerative medicine and hematological studies. This ex vivo process begins with the isolation of CD34+ hematopoietic stem and progenitor cells, which are recognized for their capacity to differentiate into various blood cell types, including erythroblasts. In a carefully controlled liquid culture environment, these CD34+ cells are exposed to a specific combination of growth factors and cytokines, prominently erythropoietin (EPO), which is essential for erythroid differentiation^4,5^. Additional factors, such as stem cell factor (SCF) and interleukin-3 (IL-3), may augment the proliferation and maturation of erythroid cells. Over time, the CD34+ cells proliferate and begin to differentiate into erythroblasts, undergoing stages of maturation characterized by changes in morphology and hemoglobin synthesis and, ultimately, loss of the cell nucleus to form reticulocytes. This ex vivo production of erythroblasts offers a valuable model for studying erythropoiesis, understanding disorders of red blood cell development, and potentially providing a source of erythroblasts for therapeutic purposes.

In addition, cell lines have traditionally played a crucial role in enhancing our understanding of erythropoiesis. The human chronic myeloid leukemia cell line K562 exhibits multipotent characteristics and can be directed to differentiate along the erythroid lineage when exposed to specific inducers that mimic physiological cues for erythropoiesis^6^. This capability makes K562 cells an invaluable model for studying various aspects of human erythropoiesis. Similarly, murine erythroleukemia cells derived from mouse erythroleukemia can also be induced and differentiated into erythroid lineage cells, providing valuable insight into the molecular mechanisms underlying erythropoiesis^7^. Another mouse cell line, G1E/G1ER cells, exhibits a conditional expression of GATA-1, a key regulator of erythroid development, facilitating studies that require precise temporal regulation of gene expression during erythropoiesis^8,9^. Despite noticeable differences between mouse and human erythropoiesis, such as the expression of fetal hemoglobin (HbF) and globin gene regulation patterns, mouse models remain indispensable. The conservation of major transcriptional regulators across species suggests that the fundamental aspects of erythropoiesis can still be effectively studied using these model systems, particularly for developmental research, genetic manipulations, and exploring in vivo interactions^10,11^.

## Genomic regulation of erythropoiesis

Regulating erythropoiesis at the genomic level involves a complex network of mechanisms directing the expression of genes essential for the development, maturation, and function of erythrocytes. Central to this regulatory system are transcription factors and epigenetic modifications, which collaborate to finely tune the expression of specific genes throughout various erythropoiesis stages. The discourse begins with an exploration of key transcription factors, underscoring their significant contributions to the regulation of erythroid gene expression and erythrocyte production. Effective gene regulation within this context requires alterations in the epigenetic landscape, notably modifications in the accessibility of erythroid lineage-specific cis elements to transcription factors. In the subsequent section, we delve into how DNA methylation, histone modifications, and chromatin remodeling impact erythrocyte development. Each component plays a crucial role in a comprehensive regulatory network that orchestrates the smooth progression of erythropoiesis, manipulating the chromatin environment to either promote or inhibit gene expression as necessary.

### Transcription factors

#### GATA-1

GATA-1 is a pivotal transcription factor in the regulation of erythropoiesis and is distinguished by its affinity for the GATA motif^12–14^, which is found in regulatory elements of all erythroid-expressed genes. It plays an essential role in activating genes vital for erythroid cell differentiation and maturation, including those encoding globins, heme biosynthesis enzymes, and erythroid cell membrane proteins^15–19^. GATA-1 is expressed in distinct lineages within the hematopoietic system, such as erythroid, megakaryocytic, eosinophil, and mast cell lineages, and its loss leads to defects in the maturation processes of erythroid and megakaryocyte populations, underscoring its pivotal role in hematopoietic lineage fidelity^12,20–22^. In addition, GATA-1 has been shown to collaborate with multiple cofactors, including FOG-1 (friend of GATA), PU.1, and p300/CBP, highlighting its central role in orchestrating the regulation of genes essential for hematopoietic lineage development and maturation processes^20,23,24^. These cooperative interactions potentially enhance its regulatory capabilities, ensuring precise control over the differentiation and maturation of blood cells.

#### TAL1/SCL

TAL1/SCL, a basic helix–loop–helix transcription factor, is crucial for early hematopoiesis. TAL1 knockout mice exhibit embryonic lethality due to an absence of hematopoiesis, and TAL1-deficient embryonic stem cells fail to contribute to any hematopoietic lineages in adult chimeric mice^25,26^. At the molecular level, TAL1 functions by dimerizing with the E proteins E12/E47 and binding to the E-box DNA motif within the promoters and enhancers of target genes, modulating their transcriptional activity^27,28^. This regulatory mechanism is essential for the proper expression of genes involved in cell proliferation, survival, and differentiation within the hematopoietic system. Moreover, TAL1 interacts with cofactors, including LMO2, GATA-1, LDB1, and RUNX1, to form transcriptional complexes that enhance its specificity and functional impact on hematopoiesis. Aberrant expression of TAL1, resulting from genetic anomalies such as chromosomal translocations, has been implicated in the development of hematologic malignancies, notably T-cell acute lymphoblastic leukemia (T-ALL)^29,30^. A comprehensive understanding of the role of TAL1 in normal hematopoiesis and its dysregulation in leukemogenesis emphasizes its importance as a potential therapeutic target in blood disorders.

#### KLF1/erythroid Krüppel-like factor (EKLF)

KLF1, also known as EKLF, is distinguished by binding to a specific DNA sequence, CCM-CRC-CCN, through its C2H2 zinc finger DNA-binding domains^31^. It is indispensable for the terminal progression of erythroid cell development and maturation and plays a pivotal role in regulating β-globin gene expression^32–34^. Targeted inactivation of the KLF1 gene in mice leads to a defect in hemoglobin production, resulting in lethal β-thalassemia, and mutations in this gene are associated with altered globin regulatory patterns^35^. In the final stages of erythroid maturation, the transition from proliferation to differentiation is carefully coordinated, partly through the regulation of the cell cycle. Transcriptomic analysis of KLF1 knockout mice revealed significant disturbances in gene networks responsible for cell cycle control^36^. In fact, the loss of KLF1 results in improper entry into the S phase during erythropoiesis, underscoring the crucial role of KLF1 in ensuring proper cell cycle progression^37^.

#### c-MYB

c-Myb, an evolutionarily conserved transcription factor, is pivotal for the proliferation, differentiation, and survival of hematopoietic cells^38–41^. Knockout of exon 6 in the c-Myb gene in mice allows the generation of primitive erythrocytes but results in defects in definitive hematopoiesis^42,43^. This observation is consistent with the results of antisense RNA experiments targeting Myb in human bone marrow mononuclear cells, further emphasizing the essential function of c-Myb in the regulation of adult hematopoiesis^44^. The expression level of c-Myb influences hematopoietic cell fate, with high levels being pivotal for erythroblast formation, directing cells toward differentiation rather than proliferation^45,46^. On the other hand, ectopic c-Myb expression is associated with hematopoietic malignancies, where continuous c-Myb expression interferes with the genetic program governing myeloid differentiation^47^. This highlights the complex role of c-Myb in supporting normal hematopoiesis and its contribution to the pathogenesis of blood cancers.

### Cis-regulatory elements

A crucial aspect of understanding erythropoiesis at the molecular level involves examining the regulatory regions that control gene expression, notably the locus control region (LCR) and the globin locus. Located upstream of the 5′ end of the β-globin gene cluster, the LCR functions as an enhancer that interacts with specific promoters of globin genes to alter chromatin structure and ensure tissue-specific and developmental stage-specific expression^48^. This region, composed of several hypersensitive sites, is essential for the proper transcriptional regulation of globin genes during erythroid differentiation. It allows the binding of various transcription factors, including GATA-1, NF-E2, and TAL1, which efficiently and precisely regulate hemoglobin synthesis and erythroid development^49–51^. This sophisticated regulatory mechanism underscores the complexity of genetic control in erythropoiesis and illustrates how uniquely programmed gene expression is fine-tuned to meet the physiological demands of oxygen transport in mammals.

### Epigenetic modifications

#### DNA methylation and histone modifications

DNA methylation has been extensively studied for its critical role in development and exhibits lineage-specific patterns in hematopoiesis^52–57^. It undergoes widespread dynamic changes, often aligning with lineage-associated enhancers and transcription factor-binding sites, highlighting the role of DNA methylation in the differentiation and maturation of erythroid cells^58,59^. Furthermore, genes implicated in DNA methylation are critical for shaping the landscape of hematopoietic differentiation^54,60,61^. The key factors involved in the DNA methylation process include DNA methyltransferases (DNMTs), such as DNMT1, DNMT3A, and DNMT3B, which are responsible for adding methyl groups to DNA, thereby modulating gene expression^52,55,60^. Additionally, the ten–eleven translocation (TET) enzymes, including TET1, TET2, and TET3, play complementary roles by catalyzing the conversion of methylcytosine to hydroxymethylcytosine, facilitating DNA demethylation^52,53,60^. This dynamic interplay between methylation and demethylation by DNMTs and TET enzymes, respectively, is crucial for the precise regulation of gene expression, impacting the differentiation and maturation of erythroid cells within the complex process of hematopoiesis.

Concurrently, histone modifications—namely, methylation, acetylation, and phosphorylation—serve as an additional layer of epigenetic regulation, influencing chromatin structure and facilitating transcriptional regulation, which is essential for erythroid differentiation^62^. These modifications modulate chromatin accessibility by either promoting transcription factor binding and gene expression or compacting chromatin to suppress gene activity. For example, histone acetylation is often associated with the activation of erythroid-specific genes, while methylation may signal either gene activation or repression, depending on the residues that are modified^63^. The key players in these processes include histone acetyltransferases (HATs) and histone deacetylases (HDACs), which are crucial epigenetic modifiers^54,64,65^. HATs add acetyl groups to histones, leading to an open chromatin structure that facilitates gene expression. Conversely, HDACs remove these acetyl groups, resulting in a more compact chromatin structure and gene repression. This dynamic interplay among HATs, HDACs, and other epigenetic modifiers, such as histone methyltransferases, is pivotal in regulating erythroid development and maturation^62,66–69^. The aberrant expression of these factors can disrupt this delicate balance, leading to hematological malignancies, underscoring the critical role of epigenetic regulation in maintaining hematopoietic health and preventing disease.

#### Chromatin remodeling

**Fig. 2:**
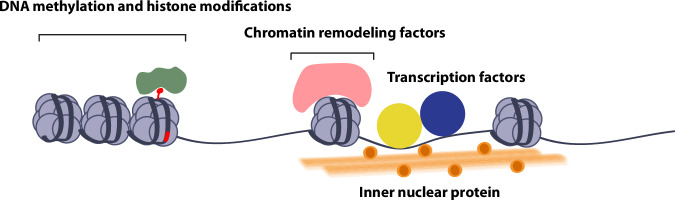
Regulation of erythropoiesis at the genomic level. Transcription factors and epigenetic modifications collaborate to precisely regulate gene expression during erythropoiesis. Chromatin remodeling factors and nuclear proteins modify chromatin architecture, making erythroid lineage-specific cis elements accessible to transcription factors. This adjustment is crucial for the orderly progression of erythropoiesis, guiding the development from HSCs to mature red blood cells.

The dynamic structuring of chromatin is fundamental for the ability of transcription factors to access cis-elements, facilitating the sequential activation and repression of gene expression, which is crucial for the various stages of erythropoiesis. Chromatin remodeling factors are pivotal in this process because they adjust chromatin accessibility to the transcriptional machinery, influencing gene expression patterns. By repositioning nucleosomes and altering chromatin compaction, these remodelers enable the precise temporal and spatial regulation of genes required for erythroid development. For example, the SWI/SNF complex, an ATP-dependent chromatin remodeler, is recruited through interactions with master transcription factors involved in erythropoiesis, altering nucleosome structure and regulating erythroid development^70–73^. Similarly, the nucleosome remodeling and deacetylation complex, in collaboration with transcription factors such as FOG-1, induces changes in chromatin accessibility, a process essential for maintaining lineage fidelity and reinforcement during hematopoiesis^74–76^. Recently, this intricate narrative of chromatin’s role in erythropoiesis has been further enriched by findings on the inner nuclear protein Matrin-3, which negatively affects cell fate transitions in erythroid cells by stabilizing chromatin organization, adding a new dimension to our understanding of the molecular mechanisms governing erythroid development^77^ (Fig. 2).

## Erythroid disorders and treatment

The transformation of HSCs to mature blood cells involves a complex, highly regulated process. At each step, a network of regulatory mechanisms ensures the precise activation of genes necessary for guiding cells to their specific destinies. Disruptions in this balanced system can lead to a range of hematopoietic disorders. For example, irregularities in red blood cell differentiation can result in inadequate development and maturation, leading to conditions such as anemia. Diamond-Blackfan anemia (DBA) is a rare congenital disorder caused by mutations in ribosomal protein genes, resulting in insufficient production of red blood cells due to defects in erythroid maturation^78,79^. Similarly, β-thalassemia is characterized by reduced or absent β-globin synthesis, leading to ineffective erythropoiesis and decreased mature red blood cell counts, thus causing anemia^80,81^.

Furthermore, altered gene expression related to cell proliferation, apoptosis, and differentiation can initiate the clonal expansion of abnormal cells, a critical step in the development of hematopoietic malignancies, including acute myeloid leukemia (AML). AML results from the transformation of hematopoietic stem or early myeloid progenitor cells in the bone marrow to malignant clones, primarily due to genetic mutations and chromosomal abnormalities^82–84^. This results in the uncontrolled growth of abnormal cells, which accumulate in the bone marrow and enter the bloodstream, thereby impairing healthy blood cell production. Erythroleukemia, an AML subtype, specifically affects red blood cell precursors, causing anemia and related symptoms^85,86^. Additionally, recent studies have shown that clonal hematopoiesis, characterized by the expansion of cells carrying specific somatic mutations, can contribute to hematopoietic dysfunction^87^. These clonal expansions, often related to age, have been reported to be associated with an increased risk of developing anemia, myelodysplastic syndrome, and AML^88–90^. This highlights the importance of maintaining delicate equilibrium in blood cell development and the significant consequences of its disruption, particularly within the erythroid lineage.

Over the past two decades, advancements in genomics, single-cell omics, and CRISPR-Cas9 technologies have significantly enhanced our understanding of the genetic factors involved in erythroid development and disorders. For instance, genomics has revealed a comprehensive landscape of gene expression and regulatory factors crucial for erythropoiesis, and single-cell omics have enabled the detailed analysis of cellular heterogeneity at various stages of blood formation^91–93^. Moreover, CRISPR/Cas9 technology represents a significant technological advance, offering not only precise functional analyses of specific genes but also therapeutic opportunities to correct genetic defects^94,95^. The utilization of these technologies has facilitated the identification and analysis of mutations associated with a range of hematopoietic disorders^96,97^. The accumulation of genomic insights plays a crucial role in both research and clinical applications, driving the development and enhancement of targeted therapies.

For genetic erythroid disorders such as β-thalassemia, DBA, and certain erythroleukemias, innovative treatments are increasingly focusing on the genetic root causes of these diseases. Gene therapy, especially through targeting the Bcl11a gene, has shown promise in mitigating symptoms of conditions such as β-thalassemia and sickle cell disease (SCD). BCL11A is integral to hemoglobin regulation and the silencing of HbF in adult red blood cells. Downregulation of BCL11A significantly benefits SCD and β-thalassemia patients by reactivating HbF production, which in turn reduces disease severity^98^. By utilizing CRISPR-Cas9 and other technologies to modulate gene expression, this approach involves modifying patients’ HSCs to decrease BCL11A expression, thereby promoting HbF production^99,100^. After these engineered cells are reintroduced into the patient, they have the potential not only to alleviate symptoms but also to offer a durable, potentially curative treatment by directly addressing the genetic anomalies driving the disorder. This strategy, underscored by the FDA’s approval of the first CRISPR gene editing therapy (Casgevy), represents a significant shift toward treating the genetic basis of erythroid disorders rather than merely managing their symptoms.

## Conclusion

The journey from a hematopoietic stem cell to a mature red blood cell encapsulates a remarkable narrative of cellular differentiation and genetic regulation. Erythropoiesis is a highly coordinated process, underpinned by the sequential activation and repression of genes, driven by a network of transcription factors, and modulated by epigenetic mechanisms. This review describes the landscape of erythroid cell generation and maturation, elucidating the complexity of regulatory pathways that ensure the efficient production of erythrocytes. The study of ex vivo erythroblast generation and the genomic regulation of erythropoiesis not only enhances our understanding of normal hematopoiesis but also sheds light on the pathogenesis of various erythroid disorders. Advances in genomic editing and gene therapy hold promise for addressing the root causes of these conditions, offering hope for the development of more effective and potentially curative treatments. As research progresses, the insights gained from studying erythropoiesis will undoubtedly contribute to the development of novel therapeutic strategies, improving outcomes for individuals affected by erythroid-related diseases. This exploration underscores the importance of continued investigation into the molecular underpinnings of erythropoiesis, paving the way for innovative approaches to combat hematological disorders and improve human health.
